# Clinical Efficacy and Safety of Injection of Stromal Vascular Fraction Derived from Autologous Adipose Tissues in Systemic Sclerosis Patients with Hand Disability: A Proof-Of-Concept Trial

**DOI:** 10.3390/jcm9093023

**Published:** 2020-09-19

**Authors:** Youngjae Park, Yoon Jae Lee, Jung Hee Koh, Jennifer Lee, Hong-Ki Min, Moon Young Kim, Ki Joo Kim, Su Jin Lee, Jong Won Rhie, Wan-Uk Kim, Sung-Hwan Park, Suk-Ho Moon, Seung-Ki Kwok

**Affiliations:** 1 Division of Rheumatology, Department of Internal Medicine, Seoul St. Mary’s Hospital, College of Medicine, The Catholic University of Korea, Seoul 06591, Korea; elwin84@hanmail.net (Y.P.); poohish@catholic.ac.kr (J.L.); wan725@catholic.ac.kr (W.-U.K.); rapark@catholic.ac.kr (S.-H.P.); 2 Department of Plastic Surgery, Yeouido St. Mary’s Hospital, College of Medicine, The Catholic University of Korea, Seoul 07345, Korea; yoonjae1116@gmail.com; 3 Division of Rheumatology, Department of Internal Medicine, Bucheon St. Mary’s Hospital, College of Medicine, the Catholic University of Korea, Bucheon 14647, Korea; sagemarple@naver.com; 4 Division of Rheumatology, Department of Internal Medicine, Konkuk University Medical Centre, Konkuk University School of Medicine, Seoul 05030, Korea; alsghdrl1921@naver.com; 5 Division of Rheumatology, Department of Internal Medicine, Incheon St. Mary’s Hospital, College of Medicine, The Catholic University of Korea, Incheon 21431, Korea; m6009@hanmail.net; 6 Department of Biomedicine and Health Sciences, College of Medicine, The Catholic University of Korea, Seoul 06591, Korea; nayakkj@empas.com (K.J.K.); dltnwlsgod1004@hanmail.net (S.J.L.); 7 Department of Plastic Surgery, Seoul St. Mary’s Hospital, College of Medicine, The Catholic University of Korea, Seoul 06591, Korea; rhie@catholic.ac.kr

**Keywords:** systemic sclerosis, stromal vascular fraction, hand disability, mesenchymal stem cells, clinical trial

## Abstract

Background: Stromal vascular fraction (SVF) has recently emerged as a potential therapeutic modality, due to its multipotent cellular components in tissue regeneration. Systemic sclerosis (SSc) is a progressive autoimmune disease that results in hand disability by skin fibrosis and microangiopathies. We performed an open-label study to investigate the efficacy and safety of SVF injection in SSc patients (Clinical Trial number: NCT03060551). Methods: We gathered 20 SSc patients with hand disability, planning for a 24-week follow-up period. SVF was extracted from autologous adipose tissues, processed by the closed system kit, and injected into each finger of SSc patients. We observed various efficacy and safety profiles at each follow-up visit. Results: Among the 20 initially enrolled patients, eighteen received SVF injection, and were completely followed-up for the whole study period. Patients received 3.61 × 10^6^ mesenchymal stem cells into each finger on average. Skin fibrosis, hand edema, and quality of life were significantly improved, and 31.6% of active ulcers were healed at 24 weeks after injections. Semiquantitative results of nailfold capillary microscopy were ameliorated. There was no single serious adverse event related to the procedure. Conclusions: Injection of SVF derived from autologous adipose tissues is tolerable, and shows clinical efficacy in SSc patients.

## 1. Introduction

Systemic sclerosis (SSc) is a progressive autoimmune disease that affects multiple internal and external organs by pathogenetic mechanisms that mainly include microangiopathies and fibrosis [1]. In spite of the high lethality and low quality of life (QOL) of the disease, to date no disease-modifying or definite curative modalities have been reported. Although recently updated treatment guidelines from the European League against Rheumatism (EULAR) covered approved treatment options based on literature reviews, only limited therapies received a high level of recommendation [2].

Theoretically, every organ could be affected through the disease progress of SSc. Hand disability is one of the most frequently observed and QOL-influencing clinical features in patients with SSc, but remains a poorly solved issue in terms of therapeutic methods, as are other systemic involvements of SSc [3]. Various phenotypes, such as Raynaud’s phenomenon, digital ulcers, skin fibrosis, and joint synovitis or contractures, are included in this category [4]. Digital ulcers and skin fibrosis of hands are regarded as the main causes of hand pain and disability [3]. Because microvascular injuries and fibrotic changes triggered by immunologic response are thought to be the biomechanics of such hand involvement, a number of pharmacologic or non-pharmacologic approaches have been suggested as treatment options [4,5]. Intravenous injection of iloprost, one of the synthetic prostanoids, is proved to have efficacy in the healing of digital ulcers, while bosentan, an endothelin receptor antagonist, reduces the additional development of digital ulcers in SSc patients [2]. However, all these treatments have various adverse effects and limitations to maintaining persistent application for long periods. Moreover, at the present time, anti-fibrotic treatment to improve skin fibrosis and joint contracture of hands is barely available [2].

To overcome such obstacles to management for hand involvement in SSc, several novel therapeutic modalities with clinical potency have been suggested. Since extraction and introduction from adipose tissue by Zuk et al. in 2002, stromal vascular fraction (SVF) consisting of multipotent cell populations, called adipose-derived stem cells (ASC), various growth factors and cytokines, has emerged as a potential therapeutic option, based on its regenerative and anti-inflammatory effects for the treatment of SSc [6,7]. According to the promising results of recent trials from French and Italian groups, long-term safety and efficacy of SVF from autologous adipose tissue were reported, improving the hand disability, QOL, and digital ulcers of SSc patients [8,9,10,11]. Moreover, a commercialized system enabling SVF to be expeditiously extracted from harvested adipose tissue makes it possible to perform whole procedures in an outpatient setting.

Herein, we report the results of an open-label proof-of-concept trial designed to investigate the tolerability and clinical outcomes of SVF administration into patients with SSc. The safety of SVF extraction from autologous adipose tissue and local injection in involved hands, and clinical efficacies in terms of SSc-related outcomes were assessed through a 24-week follow-up period.

## 2. Methods

### 2.1. Study Patients and Progress

All candidates were regarded as eligible if they were above 19 years old, and fulfilled the 2013 classification criteria for SSc [12]. Patients with uncontrolled infection in digital ulcer or significant organ involvement needing intensive immunosuppressive treatment within 6 months before enrollment were excluded. Between July 2018 and December 2019, a total of twenty subjects were enrolled in this single center, open label trial. Informed consent was obtained from all participants according to the principles of the Declaration of Helsinki. This trial was approved by the Institutional Review Board of Seoul St. Mary’s Hospital of the Catholic University of Korea (approval number: KC16CISF0365). Among the initially enrolled patients, one patient refused further procedures after baseline evaluation, while another patient dropped out due to complications during local anesthesia right before SVF injection, which is described in detail in the results section. Finally, eighteen subjects received SVF treatment, and were fully followed-up for the whole scheduled period for clinical assessment. SVF extraction and injection were performed in outpatient setting within at least 1 month after baseline evaluation. All patients were regularly assessed for intervention-related adverse events and clinical efficacies at 2 weeks (2 W), 6 weeks (6 W), 12 weeks (12 W), and 24 weeks (24 W) after their procedure.

### 2.2. Manufacture and Administration of SVF

**Figure 1:**
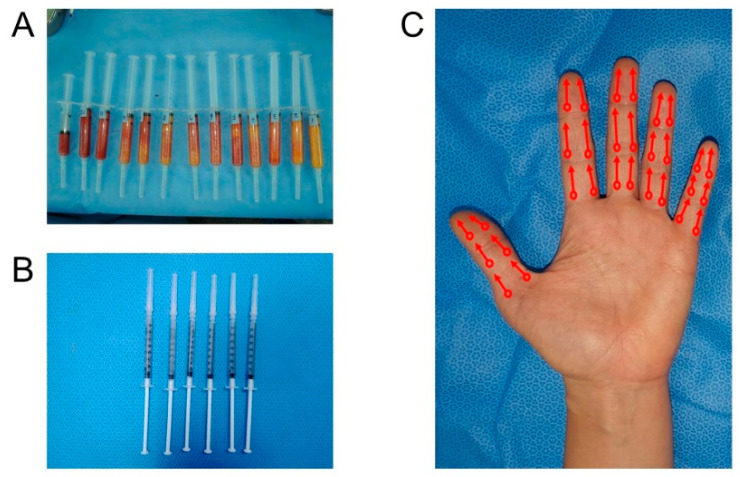
Preparation and injection of autologous fat tissue-derived stromal vascular fraction. (**A**) Harvested autologous adipose tissues. (**B**) Prepared stromal vascular fraction (SVF) mixtures for injection after the refinement. (**C**) Injection sites and needling directions of SVF mixtures. Circles indicate entry points of SVF injections, and arrows indicate directions of inserted needles using retro-tracing technique.

Fat tissue was harvested from the abdomen as the first choice, and the thigh was selected as the second choice, in the case where the amount of the fat obtained from the abdomen was insufficient. Whole harvest procedures were performed under local anesthesia, and by a suction-assisted Coleman method after tumescent solution infiltration (Figure 1A). If possible, we tried to harvest about 100 mL of fat tissue after excluding the tumescent volume, in order to obtain more cells for injection. A closed system kit (SmartX^®^ kit; DongKoo Bio & Pharma Co., Ltd., Seoul, Korea) was used to extract SVF from harvested adipose tissue. This process was performed within 50 min through repeated process of collagenase mixing, centrifuge, washing and filtering in the closed kit system. First, harvested fat tissue was treated with type 1 collagenase and incubated at 37 °C for 30 min. Then, collagenase-treated adipose tissue was carried to the component-isolating tube. This fat tissue-containing tube was centrifuged at 3000 rpm for 3 min. After that, the fat layer and supernatant were removed using a pressing unit of the kit and saline was added to the remaining bottom layer. This mixture was centrifuged at 3000 rpm for 3 min again. The washing process was repeated three times. After the washing process, the remaining SVF was extracted from the bottom of the tube. The obtained SVF, including heterogenous cells and soluble factors, was diluted to 7.0 mL, by mixing with a normal saline solution. Then 6 mL of the acquired and diluted 7 mL solution was divided into 6 syringes for injection (Figure 1B). About 15 min before the termination of SVF isolation, the fingers were blocked using 1% lidocaine by digital nerve block method. A total of 6 mL of SVF was injected into the 10 fingers of each patient, in the manner of 0.6 mL for each finger. Injection sites were located at the proximal interphalangeal joint, distal interphalangeal joint, and distal palmar crease for the long fingers, and the 3 points dividing from the metacarpophalangeal joint to the tip of the finger for the thumb (Figure 1C). Injection was performed by a retro-tracing technique, without puncturing the vessel. After gentle compression and cleansing with saline solution of the injection sites, the patient was discharged without dressing. After 20 min of treatment, hands were allowed to be used freely, and washing was also allowed. Liposuction lesions were dressed every 2–3 days, and sutures were removed 2 weeks after surgery. The remaining 1 mL was kept for biological laboratory study for cell count. Not only mesenchymal cells, but numerous cells, including red blood cell, endothelial cell, and hematopoietic stem cell, are presented in 1 mL of SVF. Therefore, in order to obtain accurate mesenchymal stem cell count results, the number of cell count was performed after SVF was applied to the plate for 12 h. Using fluorescence-activated cell sorting (FACS) and selected markers (CD73, CD13, CD34, and HLA) in adherent cells, confirmation of ASC-like cells was performed.

### 2.3. Outcomes Related to Skin Fibrosis, Hand Functions, QOL, and Digital Ulcers

The modified Rodnan skin score (mRSS, ranging from 0 to 51, with higher scores representing more severe skin fibrosis) was applied to assess the severity of overall skin fibrosis [13]. In order to evaluate the degree of hand fibrosis in particular, mRSS for hands (ranging from 0 to 12) was defined as the sums of scores of mRSS in both hands and fingers. The finger circumferences of the second to the fifth fingers measured in the middle portion of each proximal phalanx were used to assess the degree of hand edema. The Cochin hand function scale (CHFS, ranging from 0 to 90, with higher scores representing more deteriorated hand function) scores, and the Kapandji score (ranging from 0 to 10), which are both validated measures in past studies as outcomes for hand disability, were introduced in the present trial [14,15]. The severity of Raynaud’s phenomenon and hand pain was assessed using the Raynaud’s condition scale (ranging from 0 to 10, with higher scores representing more severe Raynaud’s phenomenon), and the hand visual analog scale (ranging from 0 to 10, with higher scores representing more severe pain), respectively [16]. Disease-related QOL was measured by health assessment questionnaire (HAQ, ranging from 0 to 3, with higher scores meaning lower QOL), EuroQol-5 dimensions time trade-off (EQ-5D TTO, ranging from 0 to 1, with higher values representing better QOL) values, and EuroQol visual analog scale (EQ VAS, ranging from 0 to 100, with higher scores representing better QOL) [15,17]. Digital ulcers, defined as lesions of at least 5 mm in diameter, and visible skin defects were evaluated in each finger of enrolled patients at baseline evaluation, and were regarded as healed when full epithelization occurred at any time of each follow-up, and persisted at the last follow-up visit. All the assessments were performed by clinicians who are independent of SVF procedures and certified.

### 2.4. Scoring of Nailfold Capillary Microscopic Findings

In order to assess the severity of microangiopathies in both fingers, a semiquantitative scoring system for nailfold capillary microscopic findings was adopted [18]. A total of six parameters (irregularly enlarged capillaries, giant capillaries, hemorrhages, loss of capillaries, disorganization of the vascular array, and capillary ramifications) were measured using the semiquantitative manner on the scale ranging from 0 to 3, indicating more severe as scores were higher. Each parameter was scored for four fingers of both hands, except thumbs, and the mean score value of each parameter in both hands was finally presented.

### 2.5. Statistical Analysis

Because most efficacy data consisted of non-parametric variables, results were shown as median values and interquartile range. The Wilcoxon signed-rank test was performed to analyze differences between baseline and at 2 W, 6 W, 12 W, and 24 W, respectively, using IBM-SPSS Statistics version 24.0 (SPSS Inc., Chicago, IL, USA). Statistical significance was considered at *p* < 0.05. Safety profiles were descriptively analyzed.

## 3. Results

### 3.1. Basal Characteristics of Enrolled Patients

Table 1 shows that most subjects were female (83.3%), and right-handed (100%). Although it seems a female-predominant group, considering an epidemiologic data of SSc in Korea demonstrating that the female-to-male ratio is 5:1 [19], such sex distribution in the present study could be regarded as unaffected by a selection bias. The median disease duration was 6 years. Ten patients presented as the limited type of cutaneous manifestation (55.6%). Half of the subjects received calcium channel blockers, and 13 patients were prescribed glucocorticoids, not more than 10 mg/day of prednisone or equivalent, at the time of baseline evaluation. Only one patient received an endothelin receptor antagonist. All patients showed anti-nuclear antibody positivity. Anti-centromere antibody and anti-Scl70 antibody presented in 22 and 72% of patients, respectively.

### 3.2. Acquired and Injected Cell Dose and Volume

The mean amount of adipose tissue harvested was 93.1 mL (standard deviation: ±34.4 mL) in 19 patients who finished the harvest procedure and the mean extracted SVF was 3.2 mL, as shown in Table 2. The average cell count means number of cells from 1 mL of fat tissue and the exact number was 6.08 × 10^5^. Total cells in mean harvested fat (93.1 mL) were 4.21 × 10^7^ cells. From this harvested fat, 7 cc of SVF mixtures were manufactured and 3.61 × 10^7^ cells were in 6 cc of prepared SVF mixtures. The remaining 1 cc of SVF mixtures was used for cell counting. Finally, 3.61 × 10^6^ cells were injected into each finger on average and there were 6 × 10^5^ cells in each injection syringe. Although inter-patient variability for cell yield was extensive, there were no statistically significant correlations between cell yield and involved clinical parameters. FACS data showed that 75.1 and 99.2% of adherent cells were positive for CD73 and CD13, respectively, on average, whereas 3.9 and 3.3% of cells were positive for CD34 and HLA, respectively. These results suggest that most adherent cells have ASC-like cellular characteristics. All procedures were conducted on an outpatient basis, and took up to four hours from a visit to discharge.

### 3.3. Adverse Events Related to SVF Preparation and Injection

No serious adverse events occurred during liposuction and the SVF injection process. Donor sites and injection sites were healed without infection or tissue necrosis in all patients. Five minor adverse events reported by five patients were potentially related to the procedure: one transient paresthesia in liposuction lesions, which was completely resolved in a few weeks; one patient dropout due to dizziness after lidocaine injection where the prepared SVF was discarded; and three patients in which transient pallor in fingers occurred after injection, which was resolved within 10 min after resting and warming. The pallor in fingers may be due to an increase in tension after local anesthetics and SVF injection.

### 3.4. Clinical Efficacies

**Figure 2:**
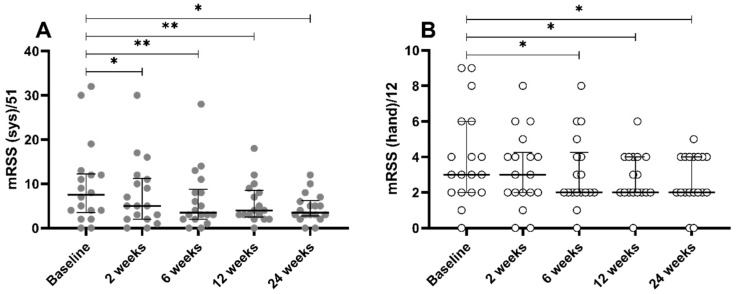
Changes of modified Rodnan skin scores. (**A**) Overall scores and (**B**) scores for hands. Bars and whiskers indicate the median values and interquartile ranges, respectively. mRSS, modified Rodnan skin score; sys, systemic. * *p* < 0.05, ** *p* < 0.01.

Overall mRSS decreased significantly from 7.5 of the median value at baseline to 5 at 2 weeks (W (*p* = 0.017)), and improvement persisted for the whole follow-up period, presenting the median value of 3.5 at 6 W (*p* = 0.001), 4 at 12 W (*p* = 0.005), and 3 at 24 W (*p* = 0.022), as shown in Figure 2A. Hand mRSS showed similar tendency of improvement, reached statistical significance at 6 W (*p* = 0.016), and also persisted until 24 W (Figure 2B). Mean circumference of both hands decreased significantly at 24 W for the dominant hand, and at 12 W for the non-dominant hand, meaning improved hand edema (Table 3 and Table 4). Disease-related QOL, represented by EQ-5D TTO values, improved by SVF injection at 24 W (*p* = 0.034), whereas EQ VAS did not reach statistical significance, in spite of the improving tendency.

Raynaud’s condition scale, related to the severity of Raynaud’s phenomenon, presented slight improvement, but with no significance (Table 3 and Table 4). The hand pain visual analog scale showed no differences through the whole follow-up period. The Kapandji score presented no change, either. The Cochin hand function scale showed increasing tendency, indicating deterioration of hand function rather than improvement, although there was no statistical significance.

Among parameters related to nailfold capillary microscopy, the parameter of “giant capillary” showed improvement at 2 W (*p* = 0.036) and 12 W (*p* = 0.013), as presented in Table 5 and Table 6. Improvement in the parameter of “irregularly enlarged capillary” at 6 W in Table 5 did not persist until the end of trial. Other parameters, such as “hemorrhages”, “loss of capillaries”, “disorganization of the vascular disarray”, and “capillary ramifications”, made no differences during the trial period.

A total of 19 cases of digital ulcers in 11 patients at baseline decreased to 13 ulcers in 7 patients at 24 W, indicating a healing rate of 31.6%. No newly developed ulcers were found in patients without digital ulcers at baseline, but eight cases of new development of ulcers occurred during follow-up periods in patients with digital ulcers at baseline evaluation, as Table 7 describes in detail.

## 4. Discussion

According to the results of the present trial, autologous fat tissue-derived SVF injection into the affected hands of SSc patients is well tolerated, and presents clinical efficacies in terms of improving skin fibrosis and QOL, healing digital ulcers, and ameliorating microangiopathies. Comparing with the results of the previous trials from France and Italy, the degree of improvements in microangiopathies, QOL and finger ulcers was relatively less but amelioration of skin fibrosis was more prominent in this trial. No single severe adverse event occurred, and several clinical improvements, such as anti-fibrotic effects and the recovery of active ulcers, occurred and did not get worse again during the period of the study.

Since the identification of undifferentiated and multipotent cell populations as its components in the early twentieth century, SVF is suggested as a new source of mesenchymal stem cells [20]. Various therapeutic approaches have been attempted and revealed as effective in terms of regenerative medicine. Because SVF is a very complex mixture of cellular components and soluble factors, exact mechanisms, including autocrine or paracrine effects, are challenging to explore. Nevertheless, because of the prompt administration without further incubation, and the ease of its acquisition compared to other sources, such as bone marrow, SVF is a promising alternative. Commercialized kits for extracting SVF from adipose tissues facilitate application to broader spectra of diseases by reducing costs and the time required for procedures.

In the present study, improvement of fibrosis, represented by decreased mRSS and mean circumferences of fingers (Figure 2, and Table 3 and Table 4), is the most prominent finding. Most past studies regarding ASC, as well as SVF, have mainly focused on their regenerative efficacy in poorly repaired wounds [6,9,10]. According to the present study, SVF treatment considerably reduce mRSS, too. Although improvement of finger edema could be regarded as amelioration of skin fibrosis, mRSS, which is validated for measurement of skin thickness through other studies, showed meaningful changes under SVF treatment. The exact mechanism of SVF is scarcely elucidated, due to its heterogeneous cell populations and various mixtures of soluble factors. Nonetheless, ASC is regarded as the most likely key factor in operating the overall effects of local SVF injection, as well as the main cause of anti-fibrotic effect [7]. Inhibiting the expression of transforming growth factor-beta is suggested as one of the modes of action of ASCs in anti-fibrosis, according to a previous study using mouse model with bleomycin-induced fibrosis [21]. Intriguingly, local injection of SVF into both hands affects the degree of fibrosis of patient’s upper arms and faces, which are distant from the initial injection sites. Although several kinds of homing molecules, which are able to reach target organs, were reported in the past studies regarding the systemic infusion of mesenchymal stem cells (MSC), the mechanisms of how locally injected SVF effects systemically remain are in need of investigation in future study [22].

Several reports in the literature have reported the regenerative potency of SVF. An Italian group performed a randomized controlled trial to compare the clinical effectiveness of adipose tissue grafting containing SVF in ischemic digital ulcers to placebo [9]. Ulcers refractory to other medical treatments were well recovered, and the number of nailfold capillaries was gradually increased for 8 weeks of follow-up periods under a single injection of SVF. Granel et al. also reported positive results regarding the recovery of digital ulcers and nailfold capillary findings in SSc using SVF injection [10]. Our results are also comparable with the previously mentioned data showing promising consequences in ischemic injuries and microscopic findings of the affected patients’ hands (Table 5, Table 6 and Table 7). Although the stricter definition of ulcer healing, defined as full recovery of skin defect, and more detailed parameters in nailfold capillary microscopic findings might compel relatively reduced responses in the present trial compared to the previous studies, SVF is also proved to be effective considering the efficacy of conventional treatment for this complication. Although the exact biological mechanisms resulting in such effects are hard to derive, several possible candidates could be speculated through previous studies. ASCs contained in SVF could promote angiogenesis in ischemic tissues by paracrine mechanisms [23]. Other cellular components, such as fibroblasts, pericytes, and several precursors cells in SVF also contribute to the regeneration of damaged tissues by secreting cytokines, and constitute the mechanical framework of repaired tissues [24,25].

Among efficacy outcomes, Raynaud’s condition scale, hand pain VAS, and CHFS showed rarely positive results in the present study. Although these variables presented significant differences, and were validated in the past studies, on the other hand, they are subjective indices that could be easily confounded by out-of-intervention factors. Seasonal variation of the severity of Raynaud’s phenomenon might influence the results of these subjective indices [26]. Nevertheless, we observed several favorable outcomes in other objective parameters. Considering a recent transcriptomic report that supports that the angiogenic and repairing capacity of SVF from patients with SSc are not compromised compared to healthy populations, and another report that shows comparable efficacy with bone marrow-derived MSC, autologous SVF in SSc patients is a reasonable alternative source of MSC and soluble factors [27,28]. In some other views, collagenase used in enzymatic treatment of SVF could lower therapeutic potency by reducing intercellular communications and may even be forbidden in the future by policies. Future studies related to SVF application should be considered in relation to these points.

The present study has several limitations to be discussed. Although this trial involved more detailed outcome measures in some aspects and a newly introduced SVF isolation method, overall results presented limited novelty except in terms of skin fibrosis compared to the past studies. In addition, cell yields of SVF showed enormous inter-patient variability in this study. This point could limit proper assessment of clinical efficacies of SVF. In some patients, aspirated adipose tissues tended to be fibrotic and scattered, and resulted in extremely low cell yield. Although such low cell yield did not statistically indicate low clinical efficacies in the present study, proper reasons for why this phenomenon occurred in SSc patients should be clarified. Furthermore, considering the absence of control groups treated with placebo and the relatively small number of enrolled subjects in this trial, further studies are in need.

## 5. Conclusions

In spite of limitations described above, this trial suggested autologous fat tissue-derived SVF as a novel treatment modality in SSc patients suffering hand disability by presenting clinical efficacy and safety in its use. During this study, there was no single serious adverse event related to the procedure and clinical improvement was noted in terms of skin fibrosis, hand edema, QOL, healing of ulcers, and microangiopathies. Future studies addressing cellular mechanisms and the ideal strategy of injection could strengthen the possibilities of further application of SVF to the treatment of SSc.

## Figures and Tables

**Table 1:** Baseline characteristics of the enrolled patients.

	Enrolled Patients (*n* = 18)
Sex (female)	15 (83.3)
Age (years)	47 (42–57)
Body mass index (kg/m^2^)	21.5 (18.2–23.1)
Disease duration (years)	6 (1–17)
Raynaud’s phenomenon duration (years)	9 (4–17)
Smoking history (current or ex-)	3 (16.7)
Interstitial lung disease	10 (55.6)
Types of cutaneous manifestations	
Diffuse	8 (44.4)
Limited	10 (55.6)
Pulmonary hypertension	1 (5.6)
History of renal crisis	1 (5.6)
Hand dominance (right)	18 (100.0)
Medical history	
Diabetes mellitus	0 (0.0)
Hypertension	0 (0.0)
Osteoporosis	6 (33.3)
Medication status	
Calcium channel blocker	9 (50.0)
Prostacyclin	2 (11.1)
Alprostadil	3 (16.7)
Endothelin receptor antagonist	1 (5.6)
Azathioprine	5 (27.8)
Glucocorticoid	13 (72.2)
Methotrexate	8 (44.4)
Ant-nuclear antibody	18 (100.0)
Anti-centromere antibody	4 (22.2)
Anti-Scl70 antibody	13 (72.2)
Anti-Ro/SSA antibody	4 (22.2)
Anti-U1 RNP antibody	5 (27.8)

Data are shown as *n* (%) or median (interquartile range).

**Table 2:** Cellular profiles related to SVF extraction.

Patient Number	Harvested Adipose Tissue (mL)	Extracted SVF (mL)	Cell Viability (%)	Total Viable Cells (× 10^7^)	Cell Yield (× 10^5^ Cells/mL of Lipoaspirate)	Injected Cells Per Each Finger (× 10^6^)
#1	80	5	98	4.00	5.00	3.43
#2	90	3	95	0.14	0.16	0.12
#3	90	2	99	0.32	0.36	0.27
#4	85	4	96	0.21	0.25	0.18
#5	90	3	94	0.09	0.09	0.07
#6	130	3	96	0.40	0.31	0.34
#7	90	3	98	0.13	0.14	0.11
#8	90	1	95	0.05	0.06	0.04
#9	70	3	97	1.80	2.57	1.54
#10	75	4	98	11.00	14.67	9.43
#11	80	2	97	1.25	1.56	1.07
#12	80	3	96	9.60	12.00	8.23
#13	90	4	97	6.16	6.84	5.28
#14	50	3	98	8.16	16.32	6.99
#15	70	2	96	12.50	17.86	10.71
#16	50	3	99	16.50	33.00	14.14
#17	160	4	98	3.00	1.88	2.57
#18	190	4	97	4.60	2.42	3.94
#19 (not injected)	110	5	96	0.12	0.11	0.10
Average (SD)	93.1 (±34.4)	3.2 (±1.0)	96.8 (±1.4)	4.21 (±5.06)	6.08 (±8.88)	3.61 (±4.34)

SVF, stromal vascular fraction; SD, standard deviation.

**Table 3:** Changes of patient-reported indices, hand function, and quality of life (2 weeks, 6 weeks).

	Baseline	2 Weeks	Changes (2 Weeks-Baseline)	*p* Value (2 Weeks-Baseline)	6 Weeks	Changes (6 Weeks-Baseline)	*p* Value (6 Weeks-Baseline)
Raynaud’s condition scale (/10)	5 (1.8–7.3)	5 (2–7.3)	0 (−2–2.3)	0.932	6.5 (4.5–7.0)	0 (−1–2.5)	0.285
Hand visual analogue scale (/10)	5 (2.5–5)	4 (2–5)	−0.5 (−1.3–0.3)	0.255	5 (3.5–6)	0 (−0.8–1.8)	0.480
Cochin hand function scale (/90)	7.5 (3.3–21.8)	14 (0–23.3)	0 (−2.5–6.5)	0.426	16 (1.5–30.3)	1.5 (−3.3–7.8)	0.172
Kapandji score (dominant hand)	9 (8–9)	9 (8–9)	0 (0–0.3)	0.234	9 (8–9)	0 (0–0)	0.334
Kapandji score (non-dominant hand)	9 (8–9)	9 (8–9)	0 (0–0)	0.257	9 (8.8–9)	0 (0–0)	0.157
EQ-5D TTO	0.807 (0.709–0.880)	0.804 (0.723–0.858)	0 (−0.034–0.090)	0.514	0.779 (0.685–0.888)	0 (−0.060–0.083)	0.906
EQ VAS	70 (50–81)	68 (50–80)	0 (−11.3–10)	0.818	70 (50–75)	0 (−18–8)	0.621
Health assessment questionnaire (/3)	1.000 (0.219–1.406)	0.938 (0.219–1.375)	0 (−0.156–0.063)	0.561	1.000 (0.000–1.563)	0 (−0.344–0.125)	0.549
Mean circumference of finger 2–5 (Right., cm)	5.9 (5.7–6.4)	5.9 (5.5–6.4)	0 (−0.1–0.1)	0.569	6.0 (5.6–6.3)	0 (−0.1–0.1)	0.552
Mean circumference of finger 2–5 (Left, cm)	5.9 (5.6–6.2)	5.8 (5.5–6.3)	0 (−0.1–0.1)	0.457	5.8 (5.5–6.3)	−0.1 (−0.2–0)	0.163

EQ-5D TTO: EuroQol-5 dimensions time trade-off; EQ VAS: EuroQol visual analog scale; VAS: visual analog scale. Data are shown as median (interquartile range).

**Table 4:** Changes of patient-reported indices, hand function, and quality of life (12 weeks, 24 weeks).

	Baseline	12 Weeks	Changes (12 Weeks-Baseline)	*p* Value (12 Weeks-Baseline)	24 Weeks	Changes (24 Week-Baseline)	*p* Value (24 Weeks-Baseline)
Raynaud’s condition scale (/10)	5 (1.8–7.3)	3 (2–5)	0 (−4–0.5)	0.228	6 (2–7.5)	0 (−2.5–0.5)	0.256
Hand visual analogue scale (/10)	5 (2.5–5)	4 (2–6)	−1 (−2–1)	0.300	5 (2.5–7)	0 (−2–2.5)	0.682
Cochin hand function scale (/90)	7.5 (3.3–21.8)	18 (1–27.5)	1 (−2.5–9)	0.255	17 (1.5–33)	4 (−2–10.5)	0.151
Kapandji score (dominant hand)	9 (8–9)	9 (8–9)	0 (0–0)	0.683	8 (8–9)	0 (−0.5–0.5)	0.516
Kapandji score (non-dominant hand)	9 (8–9)	9 (8–9)	0 (0–0)	0.276	9 (8–9)	0 (0–0)	>0.999
EQ-5D TTO	0.807 (0.709–0.880)	0.801 (0.715–0.862)	0.002 (−0.057–0.079)	0.394	0.888 (0.710–0.930)	0.058 (−0.000–0.152)	0.034
EQ VAS	70 (50–81)	80 (70–88)	5 (0–20)	0.132	80 (55–83)	0 (−7.5–22.5)	0.656
Health assessment questionnaire (/3)	1.000 (0.219–1.406)	0.750 (0.063–1.438)	−0.125 (−0.375–0.125)	0.238	0.625 (0.188–1.313)	0 (−0.500–0.063)	0.096
Mean circumference of finger 2–5 (Right., cm)	5.9 (5.7–6.4)	6.0 (5.7–6.4)	0 (−0.1–0.1)	0.740	5.9 (5.7–6.3)	−0.1 (−0.2–0)	0.019
Mean circumference of finger 2–5 (Left, cm)	5.9 (5.6–6.2)	5.9 (5.6–6.2)	−0.1 (−0.2–0)	0.015	5.9 (5.6–6.3)	−0.1 (−0.2–0)	0.050

EQ-5D TTO, EuroQol-5 dimensions time trade-off; VAS, visual analog scale. Data are shown as median (interquartile range).

**Table 5:** Changes of nailfold capillary microscopic findings (2 weeks, 6 weeks).

	Baseline	2 Weeks	Changes (2 Weeks-Baseline)	*p* Value (2 Weeks-Baseline)	6 Weeks	Changes (6 Weeks-Baseline)	*p* Value (6 Weeks-Baseline)
Irregularly enlarged capillaries	0.938 (0.594–1.969)	1.313 (0.469–1.813)	0 (−0.250–0.250)	0.975	1.000 (0.500–1.813)	−0.125 (−0.250–0.000)	0.039
Giant capillaries	0.125 (0.000–1.375)	0.000 (0.000–0.656)	0 (−0.281–0.000)	0.036	0.063 (0.000–0.688)	0.000 (−0.531–0.000)	0.057
Hemorrhages	0.000 (0.000–0.125)	0.000 (0.000–0.063)	0 (0.000–0.063)	0.495	0.000 (0.000–0.094)	0.000 (0.000–0.000)	0.671
Loss of capillaries	2.188 (1.469–2.406)	2.063 (1.438–2.500)	−0.063 (−0.281–0.156)	0.457	2.063 (1.625–2.344)	0.000 (−0.219–0.219)	0.805
Disorganization of the vascular array	0.688 (0.188–1.625)	0.219 (0.563–1.906)	0 (−0.250–0.250)	0.950	0.688 (0.000–1.688)	0.000 (−0.375–0.406)	0.753
Capillary ramifications	0.125 (0.0–0.375)	0.125 (0.000–0.438)	0 (−0.125–0.125)	0.417	0.000 (0.000–0.531)	0.000 (−0.125–0.219)	0.797

Data are shown as median (interquartile range).

**Table 6:** Changes of nailfold capillary microscopic findings (12 weeks, 24 weeks).

	Baseline	12 Weeks	Changes (12 Weeks-Baseline)	*p* Value (12 Weeks-Baseline)	24 Weeks	Changes (24 Weeks-Baseline)	*p* Value (24 Weeks-Baseline)
Irregularly enlarged capillaries	0.938 (0.594–1.969)	1.250 (0.750–1.625)	0.125 (−0.313–0.438)	0.686	1.125 (0.688–1.563)	−0.125 (−0.813–0.375)	0.372
Giant capillaries	0.125 (0.000–1.375)	0.000 (0.000–0.625)	0.000 (−0.375–0.000)	0.013	0.125 (0.000–0.313)	−0.250 (−0.938–0.000)	0.024
Hemorrhages	0.000 (0.000–0.125)	0.000 (0.000–0.188)	0.000 (0.000–0.125)	0.509	0.125 (0.000–0.313)	0.125 (0.000–0.250)	0.188
Loss of capillaries	2.188 (1.469–2.406)	2.000 (1.438–2.563)	−0.125 (−0.438–0.188)	0.333	2.250 (1.313–2.563)	0.125 (0.000–0.313)	0.372
Disorganization of the vascular array	0.688 (0.188–1.625)	0.875 (0.000–1.875)	0.000 (−0.375–0.250)	0.665	0.375 (0.000–1.938)	0.000 (−0.188–0.375)	0.633
Capillary ramifications	0.125 (0.0–0.375)	0.063 (0.000–0.594)	0.000 (−0.250–0.125)	0.504	0.125 (0.000–0.375)	0.000 (−0.375–0.125)	0.380

Data are shown as median (interquartile range).

**Table 7:** Clinical progress of digital ulcers in enrolled patients.

Patient Number	Total Ulcers at Baseline	2 Weeks Follow-Up	6 Weeks Follow-Up	12 Weeks Follow-Up	24 Weeks Follow-Up	Finally Remained Ulcers
#2	1 (R2)	ns	−1 (fully healed in R2)	ns	ns	0
#3	2 (L1, L3)	−2 (fully healed in L1 and L3)	+1 (newly developed in R5)	−1 (fully healed in R5)	+1 (newly developed in R3)	1 (R3)
#4	2 (R5, L2)	ns	−1 (fully healed in R5)	ns	+1 (newly developed in L2)	2 (L2, L4)
#5	3 (L1, L2, L4)	ns	+1 (newly developed in R3) and −1 (fully healed in L1)	ns	+1 (newly developed in L3)	4 (R3, L2, L3, L4)
#7	2 (L2, L4)	ns	ns	ns	ns	2 (L2, L4)
#8	1 (R2)	+1 (newly developed in L3)	ns	ns	−2 (fully healed in R2 and L3)	0
#9	3 (R3, L2, L4)	ns	−2 (fully healed in L2, L4)	ns	+1 (newly developed in R2)	2 (R2, R3)
#11	1 (L2)	ns	−1 (fully healed in L2)	ns	ns	0
#12	1 (R2)	−1 (fully healed in R2)	ns	ns	ns	0
#15	1 (R4)	ns	−1 (fully healed in R4)	+1 (newly developed in R4)	ns	1 (R4)
#16	2 (R2, L3)	ns	−1 (fully healed in L3)	+1 (one more ulcer in R2)	−1 (fully healed in R2)	1 (R2)
Overall	19					13

Inactive ulcers and partially healed ulcers were not counted in the “baseline” column and “follow-up” columns, respectively. Values mean number and changes of ulcers (location of ulcers, e.g., R2 means right index finger). ns—non-specific.
